# Morphology Design and Fabrication of Bio-Inspired Nano-MgO–Mg(OH)_2_ via Vapor Steaming to Enable Bulk CO_2_ Diffusion and Capture

**DOI:** 10.3390/ma15020680

**Published:** 2022-01-17

**Authors:** Hasanthi L. Senevirathna, Shunnian Wu, W. P. Cathie Lee, Ping Wu

**Affiliations:** Entropic Interface Group, Engineering Product Development, Singapore University of Technology and Design, 8 Somapah Road, Singapore 487372, Singapore; hasanthi_senevirathna@mymail.sutd.edu.sg (H.L.S.); shunnian_wu@sutd.edu.sg (S.W.); cathie_lee@sutd.edu.sg (W.P.C.L.)

**Keywords:** MgO–Mg(OH)_2_ composites, CO_2_ adsorption, hydration, electrospinning

## Abstract

The absorption of CO_2_ on MgO is being studied in depth in order to enhance carbon engineering. Production of carbonate on MgO surfaces, such as MgCO_3_, for example, has been shown to hinder further carbon lattice transit and lower CO_2_ collecting efficiency. To avoid the carbonate blocking effect, we mimic the water harvesting nano-surface systems of desert beetles, which use alternate hydrophobic and hydrophilic surface domains to collect liquid water and convey condensed droplets down to their mouths, respectively. We made CO_2_-philic MgO and CO_2_-phobic Mg(OH)_2_ nanocomposites from electrospun nano-MgO by vapor steaming for 2–20 min at 100 °C. The crystal structure, morphology, and surface properties of the produced samples were instrumentally characterized using XRD, SEM, XPS, BET, and TGA. We observed that (1) fiber morphology shifted from hierarchical particle and sheet-like structures to flower-like structures, and (2) CO_2_ capture capacity shifted by around 25%. As a result, the carbonate production and breakdown processes may be managed and improved using vapor steaming technology. These findings point to a new CO_2_ absorption technique and technology that might pave the way for more CO_2_ capture, mineralization, and fuel synthesis options.

## 1. Introduction

The increasing amount of anthropogenic carbon dioxide (CO_2_) in the atmosphere, which contributes to global climate change, has necessitated the development of new technologies and materials. Efforts to produce effective CO_2_ capture materials have been made in large numbers [1,2]. Environmentally friendly nanomaterials have recently become vital in a variety of research and development domains. Magnesium oxide (MgO) and magnesium hydroxide (Mg(OH)_2_) are two of the most environmentally friendly materials when compared to other materials [3,4,5]. For properties such as flame resistance, dielectric resistance, and mechanical strength; and micro structural properties such as porosity, large surface area catalysis, acid–base sites, and gas adsorption, MgO is well received in industrial applications such as ceramics, cement, and water treatments [2,5,6,7,8,9,10,11]. At intermediate temperatures, CO_2_ absorbents based on MgO have been identified as promising. As a result, numerous researchers have looked at using magnesium oxide to trap CO_2_. For instance, MgO-based adsorbents for CO_2_ capture produced using diverse techniques and under varied circumstances were recently evaluated by Hu et al. [1] and Ruhaimi et al. [12]. Elvira et al. [13] reported 1.61 wt.% of CO_2_ capture capacity for MgO sorbent generated by solution–combustion and ball milling at 25 °C. MgO produced using the template approach at 25 °C was examined by Bhagiyalakshmi et al. [14], who found roughly 8 wt.%. Ho et al. [15] reported 30 wt.% mesoporous MgO produced using the aerogel technique at 30 °C.

One of the most remarkable properties of Mg(OH)_2_ is its wide range of morphological shapes, which include needles, tubes, fibers, platelets, rods, and even flowers or valleys, among many other [16,17,18]. Methods for producing Mg(OH)_2_ include precipitation of a magnesium salt with an alkaline solution, the sol–gel technique, microwave-assisted approach synthesis, hydrothermal synthesis, and ammonia gas bubbling reactors. The MgO hydration approach, on the other hand, is thought to be one of the most cost-effective to date [2,19,20]. Many factors influence the hydration process, including MgO characteristics, external force environment, hydration temperature, and nucleation site. As a result, in order to obtain Mg(OH)_2_ with the desired characteristics, the reaction parameters of MgO hydration must be carefully controlled. Xing et al. [21] investigated the hydration of various active MgO under uncontrolled and ultrasonic circumstances, where the MgO hydration was governed by the main dissolving and precipitation processes at temperatures below 90 °C. Thomas et al. [22] investigated the hydration kinetics of MgO to Mg(OH)_2_ in the cement industry. The bulk of these research studies, however, does not emphasize the use of MgO hydration for CO_2_ collection.

Chemical adsorption of CO_2_ by MgO results in the formation of a MgCO_3_ layer at the surface shell, which inhibits CO_2_ molecules from diffusing into the core MgO regions through the MgCO_3_ shell. As a result, the CO_2_ collection capability is significantly lower than expected by theory. The Namib desert beetle’s effective water capture system is said to be made up of an interwoven water capture zone with high water adsorption and a water transport region with low water adsorption [23,24]. We designed our MgO–Mg(OH)_2_ composites to have an interweaved CO_2_ capture region dominated by MgO, and a CO_2_ diffusion region dominated by MgO/Mg(OH)_2_ interfaces. This was inspired by the interwoven composite. The CO_2_ that has been physically adsorbed by MgO can diffuse into the inner layers and then be chemically adsorbed by the inner MgO. Furthermore, H_2_O steam [25] may provide an additional CO_2_ diffusion mechanism. As a result, in order to collect CO_2_ efficiently at room temperature, our CO_2_ capture device employs CO_2_-philic (MgO) and CO_2_-phobic (Mg(OH)_2_) domains to mimic the water harvesting mechanism of desert beetles [23]. We used water vapor steaming at 100 °C to hydrate MgO and create Mg(OH)_2_ in electrospun MgO powders to create MgO–Mg(OH)_2_ composites. The crystal structure, morphology, and surface characteristics of the generated samples were examined using XRD, SEM, XPS, BET, and TGA. Thermodynamic and quantum mechanism models were created to aid in the analysis of the experiments.

## 2. Materials and Methods

### 2.1. Materials

For sample synthesis, analytical grade glacial acetic acid 99.8% was purchased from Scharlau (Barcelona, Spain). Analytical grade polyvinyl alcohol (PVA) (molecular weight 89,000–98,000, 99+% hydrolyzed) and Mg(OH)_2_ ≥ 99% (BioUltra) were purchased from Sigma-Aldrich (Saint Louis, MO, USA). All the chemicals were utilized without further purification. Deionized water (18 MΩ·cm) was used in all the experimental works in this study.

### 2.2. Methods

The precursor solution for electrospinning was prepared by dissolving 0.25 g Mg(OH)_2_ in 5 mL acetic acid under sonication in a water bath at 50 °C for 1 h until a clear solution was obtained. Then the aqueous PVA (5% *w*/*w*) solution, 0.750 mL, was added to the clear solution and further sonicated in a water bath at 50 °C for 30 min to eliminate any precipitation. The electrospinning was carried out in a similar manner as we reported in our earlier study [26]. The collected layer of nanofibers was kept drying at 60 °C for 48 h. The oven dried samples were then collected as solidified flakes and calcined in a muffle furnace (Nabertherm) at 350 °C for 1 h at a rate of 2 °C min^−1^ naturally cooling to room temperature. The samples were collected and ground using a motor and pestle to obtain a fine powder.

The fine powder obtained was then spread on a flat ceramic crucible and kept inside a super heating steam oven at 100 °C. Five samples were prepared by varying the steam exposure time (2-min, 5-min, 10-min, 15-min, and 20-min), and one sample was kept without exposing to steam (no-steam). The steamed samples were then kept at 30 °C for 24 h for drying.

## 3. Characterization

Surface structure and morphology were examined by using field emission scanning electron microscopy (FE-SEM) (JEOL JSM-7600F, Jeol, Tokyo, Japan). X-ray diffraction (XRD) analysis was carried out using a Bruker D8 Advance X-ray diffractometer with Cu-K radiation of 1.54. The scanning angle was adjusted from 10° to 70° with the X-ray generator running at an applied voltage of 40 kV and a current of 25 mA. Brunauer–Emmett–Teller (BET) surface area analysis was performed by using a Micrometrics ASAP 2020 system. A BET test was conducted at 120 °C using 0.1 g of powder samples. X-ray photoelectron spectroscopy (XPS) was conducted using a Thermo Fisher Scientific Theta Probe (Thermo Fisher Scientific, Waltham, MA, USA) with a monochromatic Al Kα radiation. Binding energies (BE) were determined by referencing to adventitious carbon C1s at 285.0 eV. Thermogravimetric analysis (TGA) of the samples for CO_2_ capture was conducted using a TGA Q50 analyzer (TA Instruments, New Castle, DE, USA). TGA analysis was carried out by loading 5–7 mg of samples onto a platinum pan in the TGA unit. At the beginning of the TGA run, samples were pre-treated at 150 °C for 60 min under a flow of high purity N_2_ (40 mL min^−1^) with a ramp rate of 10 °C min^−1^ to avoid errors originating from the pre-adsorbed atmospheric CO_2_, water, and other impurities. Following that, the temperature was gradually reduced to 30 °C at a rate of 10 °C min^−1^. At this stage, the gas was switched from N_2_ to high purity CO_2_ gas. CO_2_ capture capacity for all samples was recorded for 1.5 h in TGA analysis under pure CO_2_ environment. Furthermore, each sample’s TGA measurement was repeated three times to ensure that it was repeatable. The calculated standard deviation was smaller than one in the identical set of conditions.

## 4. Results and Discussion

### 4.1. CO_2_ Capture Capacity of Samples

**Figure 1:**
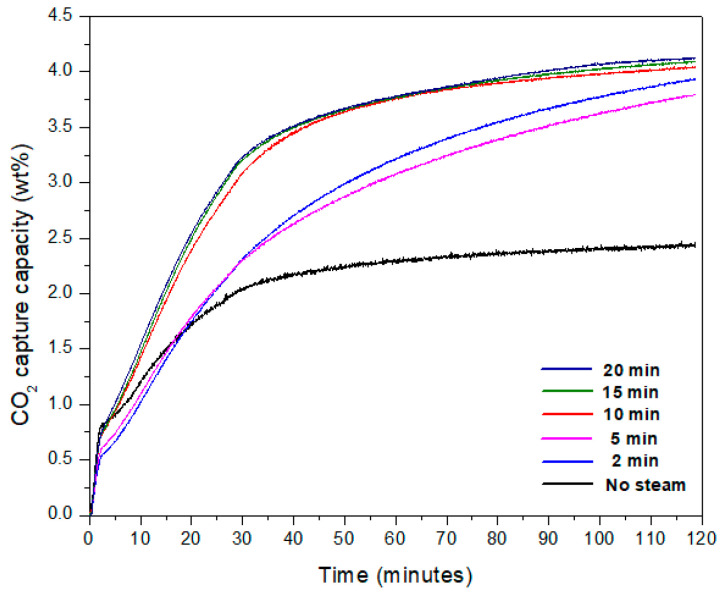
TGA data for electrospun samples before steam exposure (no-steam) and after steam exposure for different time periods (2-min, 5-min, 10-min, 15-min, and 20-min).

The CO_2_ adsorption capacity was measured using a Q50 TGA analyzer for each sample. The samples with weights in the range of 5 to 7 mg were analyzed at 30 °C with a constant flow of high purity CO_2_ for 1.5 h. The CO_2_ levels in TGA measurements approached a plateau when the testing duration reached 1.5 h, and adsorption arrived at the maximum CO_2_ capture capacity [27]. Figure 1 shows the TGA data obtained for the samples.

The CO_2_ collection capability of the samples is summarized in Figure 1. The no-steam sample was the sample as-prepared before being exposed to steam at 100 °C. We compared our outcomes before (no-steam) and after (steam) steam exposure (2-min, 5-min, 10-min, 15-min, and 20-min). Figure 1 shows that when samples were subjected to steam, CO_2_ capture was higher than when samples were not exposed to steam (no-steam sample). The CO_2_ capacity of the no-steam sample was 2.43 wt.%, compared to 4.12 wt.% for the sample subjected to steam for 20 min. As compared to the no-steam sample, the capture capacity nearly doubled after 20 min of steam exposure. The production of Mg(OH)_2_ may increase surface area, allowing for better CO_2_ collection. Furthermore, the presence of H_2_O may improve CO_2_ collection even more [25]. In addition, each sample’s data was replicated three times to ensure that it was repeatable. The computed standard deviation was smaller than 1 in the identical conditions.

### 4.2. Structural and Morphological Characterization

**Figure 2:**
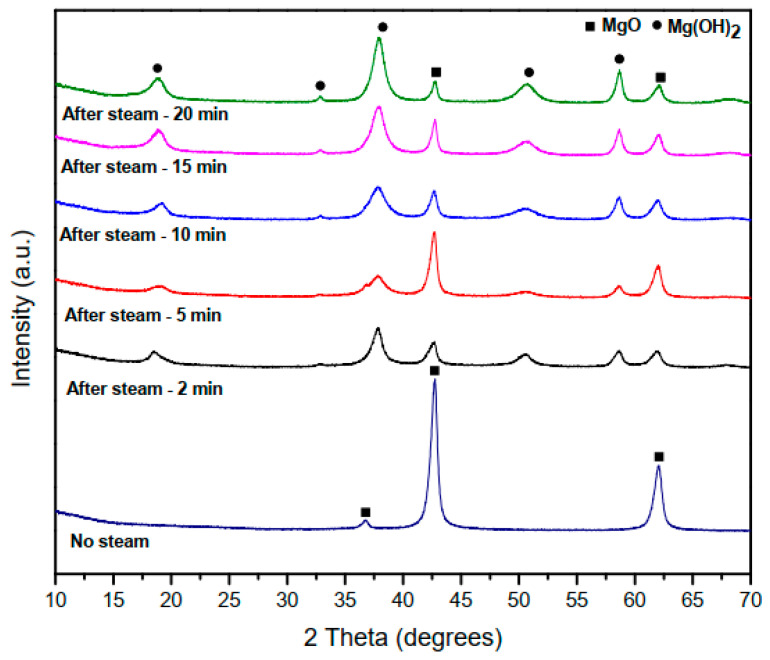
A comparison of XRD patterns for samples before exposure to steam (no-steam) and after exposure to steam for 2-min, 5-min, 10-min, 15-min, and 20-min, at 100 °C.

The structural characterization of the samples was carried out by XRD analysis. Figure 2 shows the results obtained. The results showed that the before steam sample only indicated the MgO where the main 2θ peaks of the MgO, namely 36.9°, 42.9°, and 62.3°, were consistence with (111), (200), and (220) lattice planes, respectively, were in good agreement with MgO (ICDD 00-045-0946). After the steam exposure, the peak related to Mg(OH)_2_ emerged. The steam samples showed both MgO- and Mg(OH)_2_-related peaks. The main 2θ peaks for the steamed samples of Mg(OH)_2_, namely 18.5°, 37.9°, 50.7°, and 58.6°, were consistence with (001), (101), (102), and (110) lattice planes, respectively, in good agreement with Mg(OH)_2_ (ICDD 00-044-1482). They also showed the MgO 2θ peaks 42.9° and 62.3° were consistence with (200) and (220) lattice planes. As shown in Figure 2, before the sample was exposed to steam (no-steam sample), only MgO was present, and after the samples were exposed to steam at 100 °C for 2-min, 5-min, 10-min, 15-min, and 20-min, the MgO-Mg(OH)_2_ composite structure was formed. The intensity of the peaks related to Mg(OH)_2_ increased as the steam exposure time increased, while the intensity of the peaks related to MgO decreased as the steam exposure time increased.

**Figure 3:**
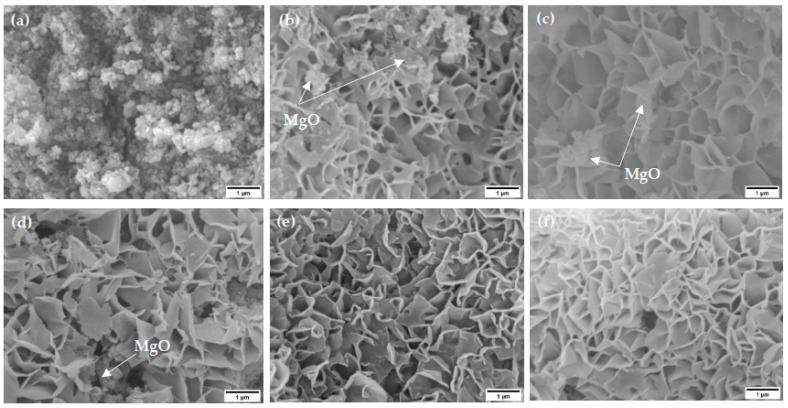
SEM images of electrospun MgO samples (**a**) before exposure to steam (no-steam) and after exposure to steam for (**b**) 2-min, (**c**) 5-min, (**d**) 10-min, (**e**) 15-min, and (**f**) 20-min at 100 °C.

The sample morphology was analyzed using SEM images, which are shown in Figure 3 below.

The sample morphology before steam exposure (no-steam) in Figure 3a shows hierarchical particle and sheet-like structures. After steam exposure, flower-like structures could be observed, as shown in Figure 3b–f. The interlaced vertical nano-sheets of Mg(OH)_2_ were firmly and uniformly grown on the samples after different time periods of steam exposure (2-min, 5-min, 10-min, 15-min, and 20-min), with an average diameter of 100 nm. The uniformity of the structures increased as the steam exposure time increased. Unreacted MgO could be observed in the 2-min (Figure 3b), 5-min (Figure 3c), and 10-min (Figure 3d) steam exposed samples along with the Mg(OH)_2_ structures, respectively. However, at 15-min (Figure 3e) and 20-min (Figure 3f), there was more uniform flower-like structure formation in the scanned area.

**Figure 4:**
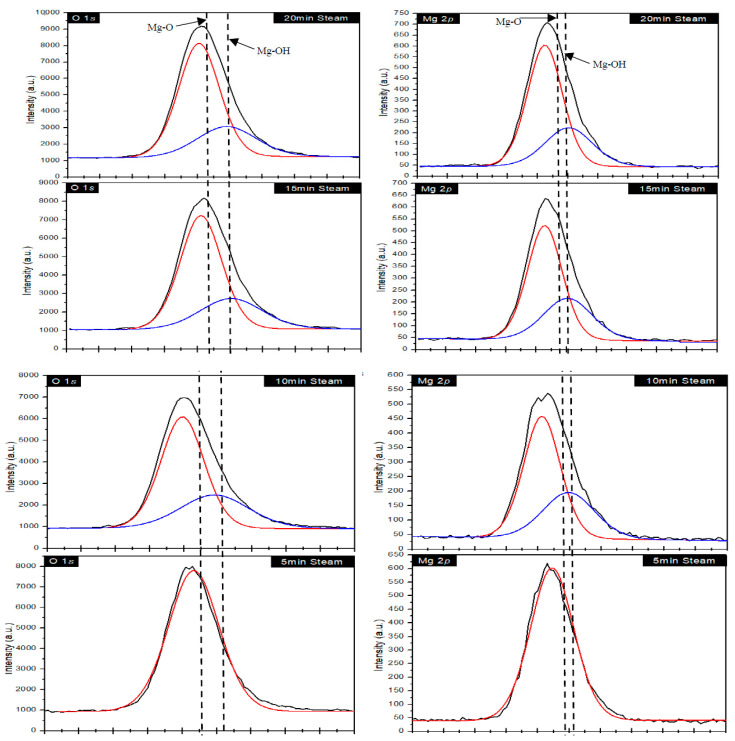

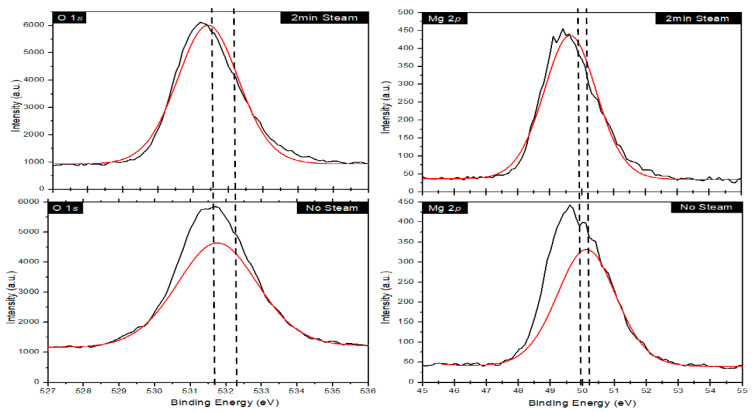
X-ray photoelectron spectra of the no-steamed and steamed samples. The no-steam sample is comprised of MgO, and the steamed samples at 100 °C show the formation of a MgO–Mg(OH)_2_ composite structure.

The XPS analysis for the samples were carried out to further analyze the surface chemistry of the samples. A summary of the data obtained for the 2-min, 5-min, 10-min, 15-min, and 20-min steam samples is shown in Figure 4.

The XPS data showed the presence of MgO and Mg(OH)_2_ in the samples. The Mg(OH)_2_ appeared in the XPS data after 10 min of steaming. The no-steam sample showed the presence of a peak located at 531.75 eV. In comparison to this energy peak, it can be seen that the peaks related to Mg–O energy decreased the binding energy with increased steam exposure time. Additionally, in both the O 1s spectra and the Mg 2p spectra, where the samples were steamed for 10-min and above, there were the presence of adsorbed –OH groups. The O 1s spectra showed that the peaks related to –OH were derived from the chemisorbed –OH groups on the surface of MgO during steaming with binding energy values of 531.89 eV, 532.03 eV, and 531.89 eV for 10-min, 15-min, and 20-min steam exposed samples. It was seen that the peak intensities in the Mg 2p spectra increased with increased steaming time, indicating the improved formation of Mg(OH)_2_ due to the chemisorption of –OH in MgO [28].

To further analyze the effect to the surface area of the samples of steam exposure, BET analysis was carried out. The surface area and pore volume parameters of samples are shown in Table 1.

Before exposing steam (no-steam) to the samples, they showed a high surface area yet a lower average pore size. This could be a reason for the low CO_2_ adsorption for the samples at 30 °C. However, once the samples were exposed to steam and with increased steaming time, the surface area of the samples showed a relative increment, which may have been due to the formation of valley-like structures observed in SEM analysis. The lower surface area of 2-min and 10-min steam exposed samples could be due to blocking of H_2_O inside the pores, which would have impaired CO_2_ chemisorption onto the MgO–Mg(OH)_2_ composites. However, Wu et al. [25] just published a theoretical analysis that shows how the presence of H_2_O considerably improves CO_2_ adsorption by MgO. In addition to the polarization caused by charge transfer from the MgO surface, the electron localization to O and C atoms inside the CO_2_ molecule results in further polarization of the CO_2_ molecule. Furthermore, the longer the sample is exposed to steam, the more Mg(OH)_2_ is produced, preventing the formation of a continuous MgCO_3_ cell, which would impede CO_2_ diffusion [29]. According to Siauciunas et al. [30], the rate of CO_2_ chemisorption is affected by the amount of water in the bermorite. At low pressure conditions, Yong and Rodrigues [31] found that a low water/steam concentration can boost the CO_2_ adsorption ability of hydrotalcite-like compounds.

## 5. Conclusions

In this research, we present an experimental study on the morphology control of MgO–Mg(OH)_2_ composite materials for effective CO_2_ adsorption at 30 °C. The experimental data show that the CO_2_ adsorption at 30 °C was improved from 2.43 wt.% to an impressive 4.12 wt.% with only 20 min by steaming time the original sample. This is due to the formation of Mg(OH)_2_, where in the study the carbonate creation and breakdown processes were controlled and optimized. These findings hint at a new method and procedure for CO_2_ absorption that could lead to new CO_2_ capture, mineralization, and fuel synthesis possibilities. By optimizing the crucial parameters such as steam duration and the calcination temperature, we aim to tune samples to improve the CO_2_ capture capacity.

## Figures and Tables

**Table 1:** Effect of hydration for surface area and pore volume parameters of samples.

Sample	Surface Area (m^2^/g)	Total Pore Volume (cm^3^/g)	Avg Pore Size (nm)
No Steam	46.76	0.156	13.35
2	8.91	0.075	33.87
5	27.67	0.160	23.17
10	14.67	0.106	28.84
15	47.02	0.300	25.56
20	52.59	0.295	22.47

## Data Availability

The data presented in this study are available upon request from the corresponding author.
